# Enhanced Mechanical Robustness of Sprayed Cellulose Nanofibril Coatings Through Internal Crosslinking with Boric Acid

**DOI:** 10.3390/polym17182451

**Published:** 2025-09-10

**Authors:** Pieter Samyn, Patrick Cosemans, Erik V. Van der Eycken, Guglielmo A. Coppola

**Affiliations:** 1 Department of Innovations in Circular Economy and Renewable Materials, SIRRIS, Gaston Geenslaan 8, B-3001 Leuven, Belgium; patrick.cosemans@sirris.be; 2 Laboratory for Organic & Microwave-Assisted Chemistry (LOMAC), Department of Chemistry, University of Leuven (KU Leuven), Celestijnenlaan 200F, B-3001 Leuven, Belgium; 3 Department of Organic Chemistry, Peoples’ Friendship University of Russia (RUDN University), Miklukho-Maklaya Street 6, 117198 Moscow, Russia

**Keywords:** nanocellulose, coating, crosslinking, boric acid, mechanical testing

## Abstract

The enhanced mechanical durability of sprayed nanocellulose coatings at the macroscopic level is primarily required to promote their application in demanding industrial applications with frequently exposed surfaces. In this study, different coating configurations are designed by spraying aqueous cellulose nanofiber (CNF) suspensions in combination with boric acid (BA) as an internal crosslinker and polydopamine (PDA) as an adhesive interlayer onto glass substrates. Multilayer coatings (CNF/BA) or mixed-layer coatings (CNF + BA) with various concentrations of BA and numbers of sprayed layers are evaluated for maximized mechanical performance based on tape tests, rub tests, cross-cut tests, and scratching tests. Good adhesive strength was realized with an interlayer of PDA/BA (high-concentration BA = 10 mM). The highest cohesive strength was observed for a mixed CNF + BA coating (high-concentration BA = 10 mM) with a scratch resistance of 9 N, and a multilayer CNF/BA coating (gradient layers with ultra-high BA concentration = 100 mM) with a scratch resistance of 8 N. The coatings with the highest density did not uniquely introduce the best mechanical resistance when comparing CNF/BA and CNF + BA coatings, as the formation of BA crystals in multilayer coatings might negatively affect the mechanical properties through embrittlement. Alternatively, the mixed CNF + BA coatings with high BA concentrations provide high density and the best mechanical resistance. The favorable crosslinking corresponds to stabilized water contact angles and reduced spreading of the water as a function of time, while a decrease in coating density causes a reduction in transparency. The chemical interactions between CNF and BA are illustrated by infrared spectroscopy, confirming a reduction in free hydroxyl groups upon crosslinking.

## 1. Introduction

With the increasing availability of nanocellulose materials at an industrial scale, these materials have become favorable candidates for common use as protective bio-based coatings. Aqueous suspensions of cellulose nanofibrils (CNFs) with appropriate viscosity and shear thinning properties allow for the homogeneous deposition of sprayed coatings on large surfaces [1]. Nowadays, nanocellulose coatings are primarily applied to create effective gas barriers against oxygen and water vapor in paper, board, and textile applications [2,3]. Alternatively, the nanoporous coating structure can be used as a host-carrier material for local integration of active species such as drugs, absorbents, or antimicrobial ingredients [4]. For more demanding conditions, however, challenges remain in optimizing their long-term performance when exposed to light, moisture, or mechanical stress [5]. In particular, mechanical robustness and durability need to be improved to enhance the coating lifetime and integrity when they are used in, e.g., manufacturing, construction, or high-touch surfaces [4]. As the coatings of native CNFs are initially formed through random organization of long fibrils, they provide intrinsic mechanical resistance depending on the mechanical entanglements and physical interactions (i.e., hydrogen bonding) between the individual fibrils. Despite the high mechanical strength of single fibrils [6], and the theoretical strength of hydrogen bonds in nanocellulose assemblies [7], however, the macroscale mechanical resistance of the sprayed coatings rather depends on the internal structural morphology developed after application [8]. Moreover, the compatibility of a nanocellulose coating with inert or mineral construction materials becomes more challenging and often requires surface grafting of silanes [9], as opposed to deposition on porous fiber-like substrates [10,11]. In particular, several methods to improve coating adhesion and stability on polymer films and textiles using physical and chemical crosslinking with polymers and polycarboxylic acids have been analyzed [12]. However, the low density and high porosity of textiles and paper are beneficial in terms of strong adhesion of the coatings as compared to flat inert substrates. Therefore, the improvement in both cohesive and adhesive properties of nanocellulose coatings needs to be addressed for high mechanical durability and robustness in daily use.

The pathways to improve mechanical, thermal, and structural stability of nanocellulose coatings by crosslinking include non-covalent (physical) or covalent (chemical) binding [13]. The mechanisms for physical crosslinking are based on charge interactions and/or hydrogen bonding, which require the additional surface modification of the nanocellulose, e.g., through the introduction of anionic carboxyl groups during TEMPO oxidation [14]. The latter often provides relatively weak properties or swelling [15]. The crosslinking of anionic nanocellulose (e.g., after oxidation) with cationic ions (e.g., H^+^ and Al^3+^) resulted in a paper coating with antimicrobial properties and reduced water absorption [16]. In composite membranes, the direct crosslinking between charged nanocellulose fibers with carboxylic groups and deacetylate chitin nanofibers resulted in better mechanical strength [17]. The reaction between the carboxylic acid and amine groups introduced during microwave irradiation also improved strength and hydrolysis resistance [18]. The crosslinking of carboxyl groups in TEMPO-CNF with polyethyleneimine (PEI) or hexamethylenediamine (HMDA) could enhance stiffness and reduce the swelling capacity of CNF foams [15]. Alternatively, crosslinking through direct interaction with the abundantly available hydroxyl groups of native nanocellulose would reduce the need for surface modification by either oxidation or grafting. Crosslinking with guar gum as a dialdehyde occurred through interaction with one or two hydroxyl groups of the nanocellulose providing mechanical reinforcement for packaging films [19], but was not transferrable to coatings due to the strong increase in viscosity under operational conditions and weak robustness in combination with the native (non-oxidized) nanocellulose fibrils [20]. Chemical crosslinking through esterification is a widely employed strategy for the modification of nanocellulose surfaces while preserving their structural integrity. This technique utilizes various reagents such as toluene sulfonic acid and hexanoic acid [21], or possible acylating agents such as butyryl, benzoyl, naphthoyl, diphenylacetyl, and stearoyl groups [22]. In recent developments, both citric acid and succinic acid were used as poly(carboxylic acid) crosslinkers for nanocellulose films [23], due to their nontoxic profile and environmental compatibility. However, the crosslinking with carboxylic acids mainly enhanced the wet strength of cellulose-based substrates [24,25], and reduced the aqueous solubility [26], while the crosslinked nanocellulose papers turned brittle after drying [25]. The most stable nanocellulose films were formed by citric acid crosslinking at high acid concentrations (>20%) in combination with heating above 120 °C, as the crosslinker simultaneously acted as a plasticizer in films with increased water vapor sorption and reduced tensile strength when no thermal heating was applied [27]. The introduction of acid crosslinkers (e.g., butane tetracarboxylic acid) as a source of anionic surface groups in nanocellulose membranes could simultaneously provide additional intrinsic properties such as conductivity, ion transport, and/or selective absorption depending on the crosslinking density [28]. After all, the chemistry of the nanocellulose coatings needs optimization during formulation and application as a higher crosslinking density may result in deteriorated mechanical properties and brittleness [29], lower biodegradability [30], or higher viscosity [31], or occupation of hydroxyl groups by crosslinking may reduce access for additional functionalization [32]. Therefore, a good balance in the degree of crosslinking should be obtained [33], or coating toughness should be altered with plasticizers such as sorbitol [34] or polyethylene glycol [35].

As an alternative environmentally benign crosslinker, boric acid (BA) is considered as a common form of boron that is naturally present in various minerals and which is nontoxic to organisms at appropriately low concentrations [36]. The favorable chemical interactions between boronic acids (a boron atom with two hydroxyl groups linked to an organic molecule) and nanocellulose were investigated, where the boron center forms covalent bonds with the hydroxylated polymer under mild conditions [37]. These bonds are reversible and influenced by the surrounding pH, allowing for a variety of applications [38]. Nonetheless, chemical modification of the polymer or molecule of interest is required to establish the boronic acid moiety. Boric acid (one boron atom linked to three hydroxyl groups) can react subsequently with two diols to form a tetrahedral complex [39], which may suggest enhanced reactivity of the latter [40]. This happens through similar mechanisms as for boronic acid and allows crosslinking within cellulose chains [41]. The interactions of borate anions with vicinal diols in the glucopyranoside moieties of cellulose could be tailored to yield chemisorbed boron chelate complexes [42], but the local cellulose backbone and side chain flexibility as well as the structures of polyol groups play a significant role in determining the kinetic and thermodynamic stability of chelate complexes [43]. Therefore, the interaction may depend on the specific morphology of the nanocellulose and its structural configuration (e.g., coatings, membranes, foams, films, or hydrogels). BA has particularly been used in crosslinking of microfibrillated cellulose composites [44], reinforcement of (nano-)cellulose particleboards [45,46,47], flame-retardant nanocellulose foams [48], nanocellulose hydrogels [49], antibacterial CNF hydrogels for drug loading [50], self-healing CNF hydrogels [51], nanocellulose adhesives [52], and food packaging films of cellulose nanocrystals and polyvinyl alcohol with antimicrobial properties [53]. The crosslinked films of hybrid CNFs and BA indeed introduced enhanced thermo-mechanical stability with additional flame retardance [54], or potentially antibacterial properties [55]. Furthermore, the addition of BA in various concentrations to nanocellulose composite coatings increased the surface hardness of coated wooden surfaces while the water contact angles declined [56]. However, the governing parameters in crosslinking of sprayed nanocellulose coatings with BA have not yet been further investigated and optimized. In combination with this, polydopamine (PDA) serves as a universal bio-inspired adhesive [57], acting as a chemical compatibilizer due to the presence of catechol groups forming complexes and chemical bonds [58]. The versatility and modulation of a self-polymerized PDA layer acting as a reactive species towards inert substrates such as glass [59] or nanocellulose [60] could substantiate its role as an adhesive interlayer. Although PDA is supposed to simultaneously serve as a crosslinker in nanocellulose hydrogels [61], its reactivity strongly depends on the oxidation state and environmental conditions (e.g., humidity, wet/dry, pH), being more difficult to control as compared to boric acid salts with a higher reactivity [62].

In this paper, the mechanical robustness of sprayed nanocellulose coatings onto glass was improved through the design of different coating configurations with either multilayers or mixed layers, in combination with boric acid (BA) as internal crosslinker and polydopamine (PDA) as adhesive interlayer. While acid crosslinkers for nanocellulose materials have been identified before, reference data for their mechanical performance as coatings is scarce. After determination of appropriate coating systems with maximized mechanical performance, the physical properties and chemical interactions between nanocellulose and BA crosslinkers at appropriate concentrations are further illustrated. Whereas most available studies relate to durability of nanocellulose coatings in textile applications, this study aims at progressing the performance of sprayed nanocellulose coatings under demanding conditions in manufacturing and construction.

## 2. Materials and Methods

### 2.1. Materials and Chemicals

The substrates to be coated were microscopic borosilicate glass slides with sizes of 25 × 75 mm^2^ (VWR Belgium, Leuven, Belgium). Dopamine (DA) hydrochloride was obtained from TCI Europe NV (Zwijndrecht, Belgium) and dissolved at a concentration of 2 mg/mL in 10 mM Tris–HCl buffer at pH 8.5 (Acros Organics, Geel, Belgium). The glass substrates were first coated with a polydopamine (PDA) layer by dipping in the DA solution for 24 h. Alternatively, the PDA coating was deposited through spraying with a solution of dopamine hydrochloride and sodium periodate (Acros Organics, Geel, Belgium). The glass/PDA substrates were thoroughly rinsed with ultra-pure water (milliQ, 18.2 μs/cm, Merck, Darmstadt, Germany) and dried at room temperature. More information on deposition of a PDA interlayer onto glass and its characterization can be found elsewhere [63,64].

The boric acid (BA) was obtained from Merck Life Science BV (Hoeilaart, Belgium) and prepared in stock solutions with three different concentrations of 1 mM (low BA), 10 mM (high BA), and 100 mM (ultra-high BA), and with the addition of one equivalent of sodium hydroxide (Merck Life Science BV, Hoeilaart, Belgium). The glass/PDA substrates were additionally coated by dipping or spraying the substrate with a low-BA or high-BA solution, followed by air drying of the glass/PDA/BA substrates.

The cellulose nanofibrils (CNFs) of Valida^®^ fine grade S191C (Sappi, Maastricht, The Netherlands) were produced as patented under industrial conditions of mechanical fibrillation at pilot scale [65], and delivered as an aqueous suspension with dry solid content of 3 wt.% (see Supplementary Figure S1).

### 2.2. Methods

The nanocellulose coating systems were deposited onto glass/PDA/BA substrates and fabricated in two configurations, of (i) multilayer coatings (CNF/BA) with alternating sprayed layers of CNF and BA crosslinker, or (ii) mixed-layer coatings (CNF + BA) with sprayed layers of a prepared CNF + BA suspension. The multilayer coatings were prepared by diluting the 3 wt.% CNF suspension to 1.5 wt.% CNF by adding demineralized water. The mixed-layer coatings were prepared by diluting the 3 wt.% CNF suspension with either the low-BA, high-BA, or ultra-high-BA solution, respectively, in a 1:1 weight ratio. The suspensions were mixed with a high-shear mixer (Orange-line Dispermill, ATP Engineering, Almere, The Netherlands) at 3000 rpm for 30 min and remained stable during spraying under selected conditions.

The coating layers were deposited with a manual air spray gun, 1000 RP (SATA GmbH, Kornwestheim, Germany), using a 1.6 mm nozzle diameter and 1.5 bar dynamic pressure after preliminary optimization of the spraying parameters. The spraying was performed in a spraying booth with controlled airflow and a temperature of 25 °C. The glass substrates were mounted on a horizontal table with perpendicular orientation of the spray gun at 20 cm above the samples, while applying a horizontally advancing gun speed of 10 mm/s. The multilayer coatings and mixed-layer coatings were sprayed in thicknesses corresponding to 3, 5, and 10 layers of CNF with alternating BA layers (multilayers), or 3, 5, and 10 sprayed layers of CNF with mixed BA (mixed layers). The respective layers were deposited in “wet” conditions (no intermediate drying in between the sprayed layers, final drying for 30 min at 70 °C after spraying the final CNF top layer) or “dry” conditions (with intermediate drying after each deposited layer for 30 min at 70 °C in a circulating hot-air oven). The samples were stored under a conditioned atmosphere (23 °C, 50% RH) before further characterization.

### 2.3. Characterization Techniques

The mechanical resistance of the coatings was evaluated by a micro-scratching test using the sclerometer type 3092 (Elcometer Instruments GmbH, Aalen, Germany) with a tungsten carbide tip of 0.75 mm radius loaded under 0 to 11 N by exchange of the spring with an appropriate spring constant, following ISO 1518-1 [66]. The stylus travelled at a speed of 35 mm/s over a length of the scratching track of 40 mm. The cross-cut testing was applied according to ISO 2409:2020 [67], using the cross-hatch adhesion tester type 1542 (Elcometer Instruments GmbH, Aalen, Germany) with 2 mm cutter spacing and adhesive tape. The performance results are quantified by scoring grades 0 (good adherence) to 5 (failure). The tape test was performed on either a native coating or after cross-cut testing by peeling in a 90° perpendicular direction. A fingernail rubbing test was performed by scratching one stroke in agreement with the DBL 7383 specifications. Microscopic evaluation of the mechanical damage was performed through optical microscopy on a Keyence VHX-7000 (Keyence, Mechelen, Belgium) with ×20 to ×200 magnification of objective lenses.

The detailed morphologies and 3D topographies were studied on a confocal laser scanning microscope VK-X3000 (Keyence, Mechelen, Belgium) with ×10 to ×150 objective lenses (additionally, with up to ×8 digital magnification). The surface roughness parameters (Sa = average surface roughness) were determined on images at ×20 or ×50 magnification, after a consistent surface flattening procedure eliminating the long-range waviness, following ISO 25178 [68]. The coating thickness was determined as an average of 10 height differences measured from line scans over a cross-sectional scratch at ×20 magnification. The weight of the different coating layers was measured on an analytical balance (Sartorius, Göttingen, Germany) with 0.001 mg precision.

The static water contact angles were determined with an OCA50 goniometer (Dataphysics Instruments GmbH, Filderstadt, Germany) after depositing a 3 µL droplet of deionized water and shape fitting with a tangent fit procedure for hydrophilic surfaces according to ISO 19403-2 [69]. The evolution of the contact angle during spreading of the water droplet over the surface was followed for 20 s. UV/VIS spectroscopy was performed on a UV-2450 spectrophotometer (Shimadzu, Kyoto, Japan) in transmission mode recording the transmission spectra over the 200 to 900 nm wavelength range, relative to a calibrated reference material of a pure (non-coated) glass slide.

Fourier transform infrared spectroscopy (FTIR) was performed in attenuated total reflection (ATR) mode on a Nicolet iS10 spectrometer (Thermo Fischer Scientific, Breda, The Netherlands) equipped with a diamond crystal and via sampling at 4 cm^−1^ resolution, with 16 scans between wavenumbers of 4000 and 500 cm^−1^.

## 3. Results

### 3.1. Mechanical Testing of Coatings

**Figure 1:**
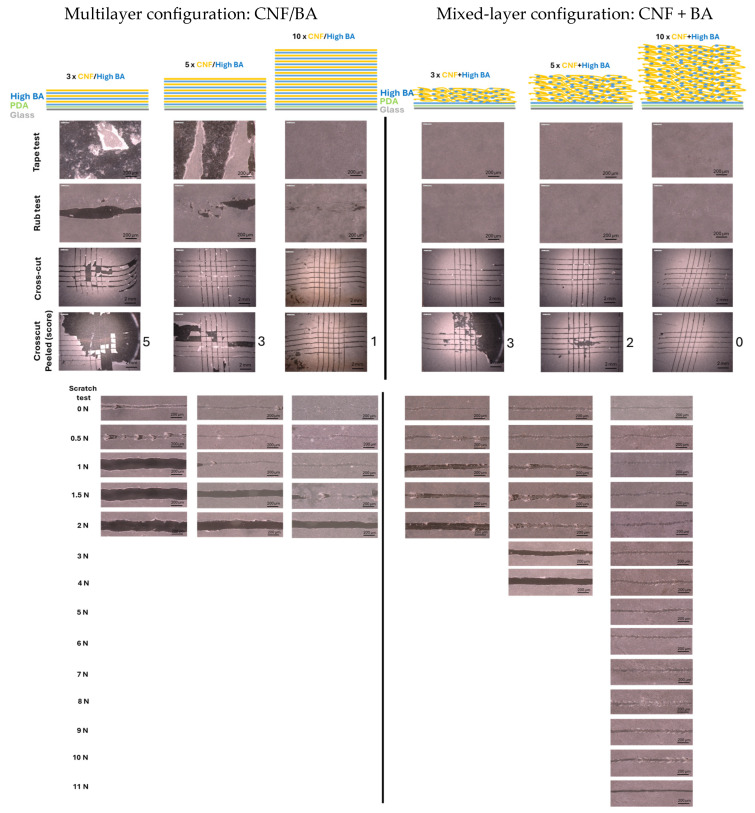
Mechanical resistance testing of multilayer (CNF/BA) coatings (**left**) and mixed-layer (CNF + BA) coatings (**right**), with the same high-BA crosslinker concentrations (10 mM) in the interface layer and coating bulk.

**Figure 2:**
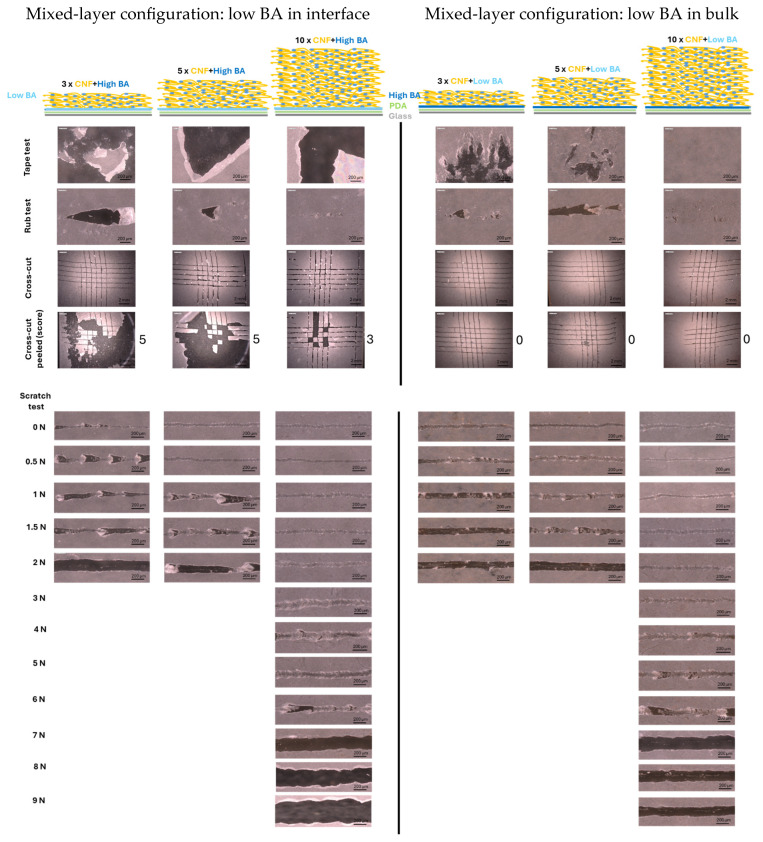
Mechanical resistance testing of mixed-layer (CNF + BA) coatings with low-BA crosslinker concentrations (1 mM) either in the interface layer (**left**) or in the coating bulk (**right**).

**Figure 3:**
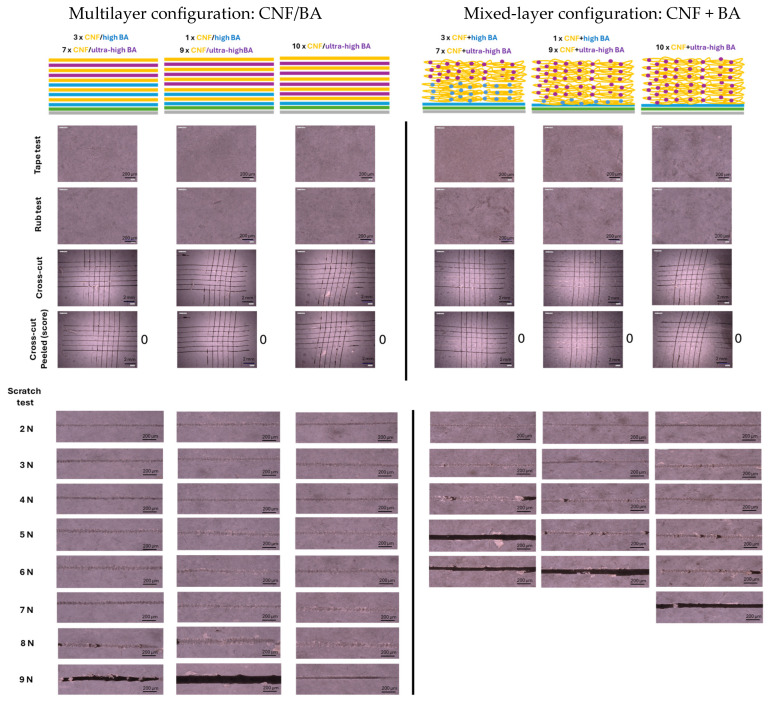
Mechanical resistance testing of multilayer (CNF/BA) coatings (**left**) and mixed-layer (CNF + BA) coatings (**right**) with ultra-high-BA crosslinker concentrations (100 mM) in the interface layer and coating bulk, applied in combination with the high-BA crosslinker concentration (10 mM), creating gradient coating layers.

The mechanical properties of sprayed nanocellulose coatings strongly rely on the formation of a homogeneous and dense coating with internal hydrogen bonding, as evaluated previously [70]. However, the better mechanical properties of surface-modified nanocelluloses also indicated that additional crosslinking is needed for improved robustness and durability in practical applications [70]. Fulfilling the requirement to formulate nanocellulose coatings with enhanced mechanical resistance through crosslinking with BA, the macroscale mechanical properties of nanocellulose coatings with different configurations, layer thicknesses, and BA concentrations were evaluated. The multilayer (CNF/BA) coatings and mixed-layer (CNF + BA) coatings were deposited with 3, 5, and 10 spraying layers of nanocellulose, while evaluating the influences of coating configuration (Figure 1), low BA concentration in the interface layer or bulk of mixed-layer coatings (Figure 2), and high BA or ultra-high BA concentration in the bulk of multilayer or mixed-layer coatings (Figure 3).

The results for mechanical testing of multilayer versus mixed-layer coatings (Figure 1) were evaluated with optical micrographs after a tape test, rub test, cross-cut test without and with peeling (scoring grade 0 to 5), and scratch test under different normal loads. The reference coatings with pure nanocellulose (no BA) sprayed in 3, 5, and 10 layers were not further evaluated as they yield a low scratch resistance of <0.5 N. As a preliminary reference test for evaluating the role of pretreatment on the glass substrate, the coatings deposited onto bare glass substrates showed immediate adhesive failure: the presence of an adhesive interlayer of PDA and a first BA crosslinking layer on the glass/PDA/BA substrates was preferred to improve the wetting of the glass substrate and introduce adhesive bonding. For both multilayer (CNF/BA) and mixed-layer (CNF + BA) coatings, the high BA concentrations were initially used as a first crosslinking layer on top of the PDA adhesive interlayer on the glass interface, while simultaneously using the same high BA concentrations as a crosslinker in the bulk of the nanocellulose coatings. For the multilayer coatings with a high-BA crosslinker (CNF/high BA) (Figure 1, left), mechanical resistance is relatively weak with immediate mechanical failure for the multilayer coatings with 3 and 5 sprayed layers and somewhat better mechanical resistance for the multilayer coatings with at least 10 sprayed layers. However, the proposed multilayer coatings with a high-BA crosslinker did not provide a good combination of adhesive and cohesive strength, as failure immediately occurred after rub testing and scratch testing at 2 N for CNF/high BA (10 layers). For the mixed-layer (CNF + high BA) coatings with a high-BA crosslinker (Figure 1, right), better mechanical performance is noticed with a progressive increase in mechanical resistance with a higher number of sprayed layers, up to a maximum scratch resistance of 9 N (10 layers). The fracture pattern of the high-BA-crosslinked nanocellulose coatings is characterized by chevron lines behind the scratching track, which typically indicate a rather brittle failure of the coatings. These can intuitively be attributed to the formation of a densely crosslinked nanofiber network with low mobility of the single nanocellulose fibers (see below). Alternatively, the failure pattern of the coatings with weak mechanical resistance shows more deformation in the scratching track with the local upsetting of coating fragments in the scratching track and finally full coating removal. The local bulging of the coating implies interfacial failure in combination with local sliding effects of the coating over the glass substrate under scratching. The broad scratching tracks in the mechanically weak coatings are consequences of tearing apart of the loosely interlocked nanofibrils in the coating. In contrast with the presented coating configurations including intermediate drying of the subsequently deposited layers in multilayer or mixed-layer coatings, the coatings without intermediate drying between the different layers developed lower mechanical resistance (see Supplementary Figure S2). Therefore, intermediate drying after each sprayed layer better contributes to crosslinking for both multilayer and mixed-layer coatings. This could be explained by a higher degree of borate ester formation. The boric acid molecule needs to undergo two subsequent condensation reactions to form a stable crosslink between cellulose fibers, and water is produced as a byproduct [40]. When heat is applied it accelerates the loss of water molecules from the coating, and therefore the reaction equilibrium is pushed to completion. An incomplete reaction, perhaps in the absence of a drying step, would result in the instauration of weaker hydrogen bonds between the hydroxyl groups of the borate and cellulose. As an alternative method for deposition of the PDA/BA adhesive interlayer by dipping, the PDA and high-BA interface layers were sprayed and the coating performances were compared relative to the coatings with a single PDA interface layer without high BA (see Supplementary Figure S3); however, the lower coating adhesion in the absence of a high-BA interface layer (5 N scratch resistance, compared to 9 N) and lower mechanical properties of the coatings with a sprayed PDA/high-BA interface layer (8 N scratch resistance) were confirmed, as compared to a dip-coated high-BA interface later (9 N scratch resistance). The mechanical performance of the coating system indeed relies on the homogeneity (e.g., density) and crosslinking of the interface layer on glass as demonstrated by further chemical and physical characterization (see Section 3.4). While the present study focusses on the increase in robustness of coating systems with multilayers or mixed layers, the role of the single adhesive PDA or PDA/BA interface layer will be fully detailed in a separate study indicating negative influences due to higher roughness and porosity of a sprayed high-BA interface layer.

Starting from the same high BA concentrations as a crosslinker in the interface (glass/PDA/BA) and within the bulk of the CNF + BA mixed layers, the effects of low BA concentrations in either the interface or the bulk of the mixed-layer coatings were further evaluated (Figure 2). As a reference system, the preliminary adhesion trials with a single nanocellulose layer sprayed on pure PDA (no BA crosslinker in the interface) indicated immediate adhesive failure of the nanocellulose coating. Therefore, the synergistic effects between PDA and BA in the interface enhance the adhesion of the nanocellulose layers and can be explained by the formation of heteroleptic boron complexes with both the catechol units of PDA and the sugar residues from nanocellulose. Herein, the asymmetric borate ester acts as a linker between nanocellulose and the PDA layer, and is strongly adhered onto the glass surface. However, when a first layer of low BA concentration is applied in the interface in combination with a high BA concentration as a crosslinker in the bulk (Figure 2, left), tape test and cross-cut analysis indicate that the coating is more sensitive to adhesive failure in the interface with clear delamination as compared to the coatings with a high-BA interface layer. This corresponds with a reduced scratch resistance of 7 N and full removal of the coating from the substrate within the scratch track. Therefore, glass/PDA/BA interfaces with low BA concentration are not sufficient for good adhesion as compared to those with high BA concentration. While reducing the BA concentration as a crosslinker in the bulk of mixed-layer coatings, the coatings with mixed CNF + low-BA layers (Figure 2, right) present lower mechanical resistance than the coatings with mixed CNF + high-BA layers: the mixed CNF + low-BA coatings have a maximum scratch resistance of 6 N as compared to the scratch resistance of 9 N for mixed CNF + high-BA coatings. The mechanical failure of the coatings with low BA crosslinker concentrations is mainly attributed to cohesive failure, indicating a lower degree of crosslinking. As demonstrated from the tape test and cross-cut test, however, the adhesive properties of the mixed-layer (CNF + low BA) coating with 10 layers are maintained with a high-BA interface layer. Although the internal strength of the coatings with low-BA crosslinker concentrations in the bulk fail, the good adhesive properties remain controlled by the high BA concentration in the interface. As such, mechanical resistance in both the bulk and the interface of the coating are separately controlled by the BA concentration.

Finally, the mechanical properties with ultra-high-BA crosslinker concentrations in the bulk of the coatings were evaluated for multilayer (CNF/ultra-high BA) and mixed-layer (CNF + ultra-high BA) coatings (Figure 3). Also, the application of an ultra-high BA concentration in the adhesive interlayer glass/PDA/BA was investigated, but it immediately caused degradation of the PDA layer. This effect can be associated with a strong interaction of the catechol units with boric acid, which was reported as an effective inhibitor of dopamine polymerization and PDA adhesion [71]. Dip coating with the ultra-high BA concentration caused substantial leaching from the PDA layer, observed as discoloration of the brown coating and complete detachment after a few hours. For this reason, the gradient coating configurations were designed with the initial layers with high BA concentrations in contact with the glass/PDA interface layer, while avoiding direct contact between the glass/PDA interface layer and the ultra-high BA concentrations. In combination with a high-BA interface layer, a number of intermediate high-BA layers were applied in both multilayer coatings and mixed-layer coatings. For the multilayer coatings (Figure 3, left), a single high-BA interface layer in contact with the glass/PDA adhesive layer efficiently provides good mechanical adhesion with the substrate. The cohesive strength of the coating under scratch testing has improved towards 8 N for multilayer coatings with CNF/ultra-high BA in contrast with the scratch resistance of 1.5 N for multilayer coatings with CNF/high BA. Adversely, for the mixed-layer coatings (Figure 3, right), good adhesive strength is provided through the high-BA interlayer while the cohesive strength is reduced towards a maximum scratch resistance of 6 N for the mixed-layer (CNF + ultra-high BA) coatings in contrast with the scratch resistance of 9 N for the mixed-layer (CNF + high BA) coatings. This can intuitively be attributed to the very dense crosslinking of the mixed-layer (CNF + ultra-high BA) coatings with reduced mobility of the entangled fiber network and high brittleness, as visually observed by the chevron lines in the scratching track. In contrast, the mixed-layer (CNF + high BA) coatings with the highest scratch resistance show a smooth scratching track related to a ductile failure mode.

In conclusion, a balance between cohesive and adhesive failure for dried coatings with enhanced mechanical robustness has been experimentally determined through controlling the BA crosslinker concentration in the glass/PDA/BA interface and bulk of the coating. Depending on the coating configuration, the multilayer (CNF/ultra-high BA) coatings and mixed-layer (CNF + high BA) coatings show the best cohesive properties, while an interface layer with high BA concentration is required for enhanced adhesion. The chemical interactions in relation to qualitative data for crosslinking, confirming the enhanced mechanical properties, are further discussed in the following paragraphs.

### 3.2. Morphology and Surface Roughness

**Figure 4:**
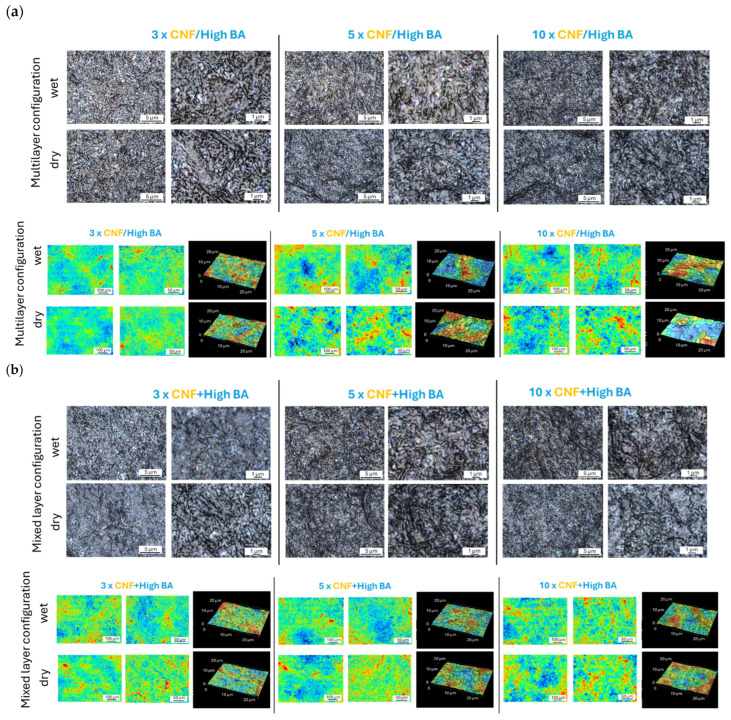
Morphology and topography images obtained from confocal laser scanning microscopy for different coating configurations deposited under wet conditions (no intermediate drying) or dry conditions (with intermediate drying) with different numbers of subsequently sprayed layers in (**a**) multilayer (CNF/BA) coatings and (**b**) mixed-layer (CNF + BA) coatings. Color figures represent z-scale from blue (0 µm) to red (5 µm).

The morphology and topography of sprayed nanocellulose coatings with high BA crosslinker concentrations in different configurations are illustrated in Figure 4, including the multilayer (CNF/high BA) coatings (Figure 4a), and mixed-layer (CNF + high BA) coatings (Figure 4b) deposited with intermediate drying or without intermediate drying (wet). Within a homogeneous coating region of fiber deposits, the microscopic images at different magnifications (×600, ×1200) show a regular arrangement of CNF nanofibrils and formation of a densely packed nanofiber coating with an intrinsic nanoscale pattern related to the organization of the nanofibrils. The 2D and 3D topographical images at different magnifications (×20, ×50, ×600) show the progressive formation of fiber entanglements and interactions as the coating thickness increases. With an increasing number of sprayed layers, there is a tendency for fiber agglomeration and formation of protrusions at the surface to occur.

**Figure 5:**
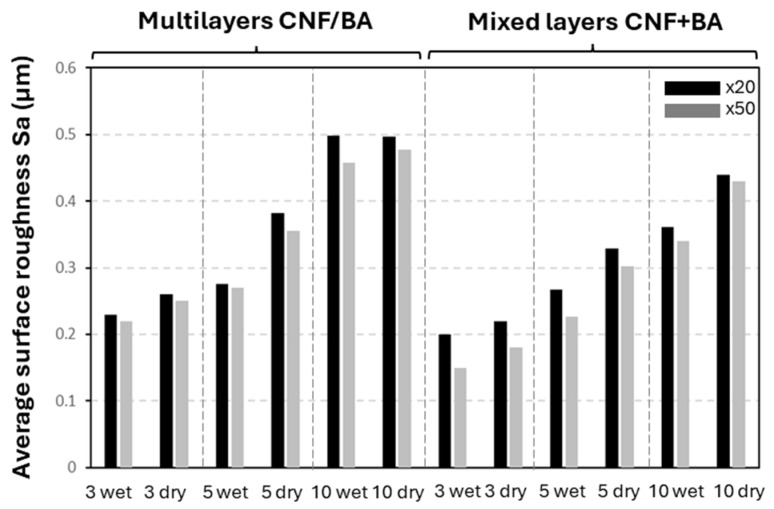
Average surface roughness values Sa for multilayer (CNF/BA) and mixed-layer (CNF + BA) coatings, with different numbers of subsequently sprayed layers deposited under wet conditions (no intermediate drying) or dry conditions (with intermediate drying).

The values for average surface roughness Sa were determined from the topographical surface scans at a magnification of ×20 or ×50, indicating the differences between multilayer (CNF/high BA) coatings and mixed-layer (CNF + high BA) coatings (Figure 5). The changes in surface roughness for different coating configurations and coatings with different numbers of layers are consistent for both magnifications, demonstrating a good representation of the coating morphologies at different scale levels. The average surface roughness Sa obviously increases progressively with a higher number of sprayed layers, while it is consistently higher for the coatings with intermediate drying (as compared to a wet deposited layer), and lower for the mixed-layer coating layers (as compared to the multilayers). The results indicate that the multilayer coatings are more heterogeneous as compared to the mixed-layer coatings, while additional structural organization or crosslinking during the intermediate drying steps may affect the arrangement of the nanofibrils, resulting in a higher surface roughness. The lower surface roughness of the mixed-layer coatings compared to multilayer coatings may suggest fewer fibril protrusions at the surface and a lower porosity, in parallel with a higher crosslinking density. Also, the intermediate evaporation of water during intermediate drying may introduce local re-arrangements and organization of the nanofibrils. As compared to the sprayed coatings of CNF (no BA crosslinker) in three or five layers [70], the values for average surface roughness are in the same range, between Sa = 0.2 µm (three-layer CNF) and Sa = 0.4 µm (five-layer CNF). The roughness of sprayed CNF coatings shows good agreement with the roughness of mixed-layer (CNF + high BA) coatings, while the higher roughness of multilayer (CNF/high BA) coatings indicates their more heterogeneous morphology.

**Figure 6:**
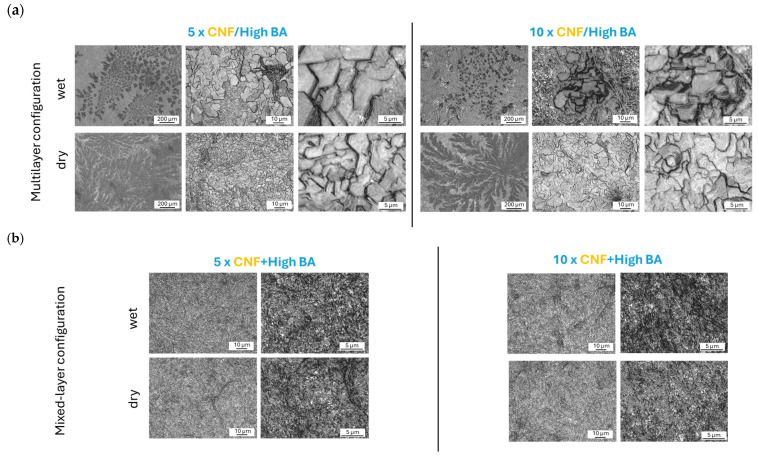
Detailed morphological evaluation of coating homogeneity/heterogeneity, indicating (**a**) the local formation of BA crystallites in the case of multilayer (CNF/BA) coatings, and (**b**) homogeneous fibrous morphology in the case of mixed-layer (CNF + BA) coatings.

The more detailed observations of local coating morphologies at different magnifications, as shown in Figure 6, indicate the presence of organized crystalline structures in multilayer coatings (both CNF/high BA and CNF/ultra-high BA). Depending on the drying conditions, the crystallite shapes are more spherical for the wet layer deposits, while they have further developed into dendrite shapes after intermediate drying. The more specific details on different crystallite structures for multilayer coatings with or without intermediate drying are shown in Supplementary Figure S4. The crystallite formation under drying of BA from the aqueous phase has been described before and results in similar platelet structures, where the crystals are formed by dehydration under evaporation and the crystallite size depends on the concentration, dispersant, temperature, and evaporation rates of BA [72]. If present, a secondary phase such as nanocellulose may either act as a nucleator or a crystal growth inhibitor during the crystallization of BA, depending on its miscibility and surfactant properties [73]: in particular, BA crystals with a hexagonal shape were obtained in combination with other solvents and have the most developed shapes after drying at around 70 °C in the presence of carbohydrates [74]. For mixed-layer (CNF + BA) coatings, no crystallization effects were observed as the nanofiber network likely hinders crystal growth, resulting in more homogeneous coating morphologies. In relation to the observed coating performance, the crystallite formation in multilayer (CNF/BA) coatings obviously weakens the mechanical properties as it might introduce brittleness or reduce the interactions between BA and CNF. The formation of BA crystals as contaminants also occurred previously under conditions of relative humidity above 15% and resulted in poor adhesion properties of multilayer packaging due to the increase in softening temperature [75].

**Figure 7:**
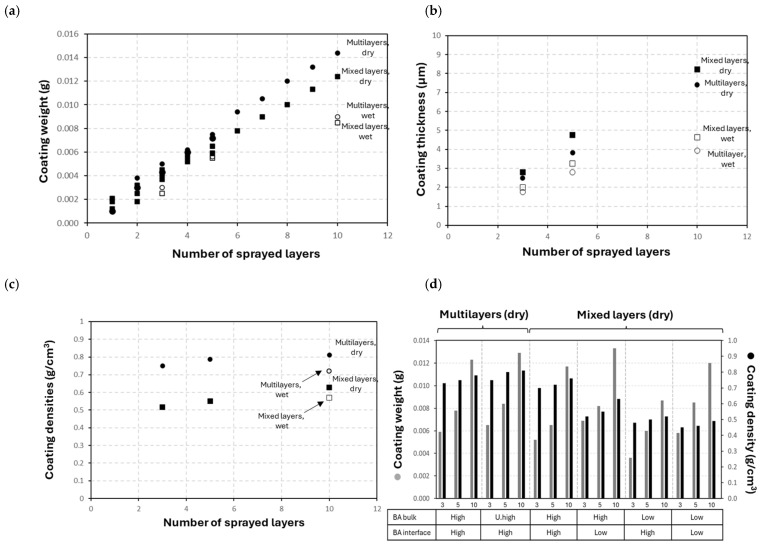
Coating deposition characteristics as a function of the number of sprayed layers and final characteristics, including (**a**) coating weight, (**b**) coating thickness, and (**c**) coating densities. (**d**) Coating weight and coating density summary for different coating configurations with 3, 5, and 10 sprayed layers.

The coating thickness and density for multilayer and mixed-layer coatings are illustrated in Figure 7, as determined from experimental weight and thickness measurements. There is a linear increase in coating weight with a higher number of sprayed layers (Figure 7a) for both the multilayer (CNF/high BA) and the mixed-layer (CNF + high BA) coatings. The repetition of the measurements is confirmed through good overlap in weight data for the coatings with 3, 5, and 10 sprayed layers and intermediate drying for both multilayer and mixed-layer coatings. The weight of multilayer (CNF/high BA) coatings is somewhat higher as compared to the mixed-layer (CNF + high BA) coatings, likely because of the lower control on subsequent spraying of the two separate layers of CNF and high BA with a slight overload of BA (higher than the 1:1 ratio as compared to the mixed-layer coatings). Otherwise, the increase in weight of the wet deposited multilayer and mixed-layer coatings does not follow a consistent trend, deviating from a linear increase (mainly at a higher number of sprayed layers) due to an overspray with partial removal of the wet coating layer upon subsequent spraying. As opposed to the weight measurements, however, the coating thickness for the mixed-layer (CNF + high BA) coatings is higher than that for the multilayer (CNF/high BA) coatings (Figure 7b). Therefore, the calculated coating densities (Figure 7c) indicate higher densities for the multilayer (CNF/high BA) coatings, and lower densities for the mixed-layer (CNF + high BA) coatings. As a function of BA crosslinker concentrations in the bulk or interface layer (Figure 7d), the coating densities were calculated from coating weight and thickness measurements. In comparison, the coatings with a sprayed PDA/BA interface layer on glass demonstrate inferior properties and were not further considered (see Supplementary Figure S5); in particular, the low density of the coatings with a sprayed interface layer indicates a higher heterogeneity, in line with the lower mechanical resistance of these coatings as observed before.

In parallel with previous data for spraying of CNF coatings [69], the coating density increases with the number of sprayed layers and stabilizes at above five to six sprayed layers due to the presence of local heterogeneities for the thinner coatings. For the multilayer coatings, densities are higher as compared to the mixed-layer coatings, and the increase in crosslinker concentration introduces an increase in coating density, shown by comparing the multilayer coatings (CNF/high BA and CNF/ultra-high BA). The higher density for multilayer coatings can be attributed to formation of crystallites, as demonstrated in the previous paragraph. For the mixed-layer coatings, the higher BA crosslinker concentrations also involve an increase in coating density by comparing mixed-layer CNF + low-BA and CNF + high-BA coatings, which confirms the crosslinking in the bulk of the coatings.

In relation to the reported mechanical performance of multilayer and mixed-layer coatings, the coating configurations with the highest density do not uniquely introduce the best mechanical resistance when comparing multilayer (CNF/BA) and mixed-layer (CNF + BA) coatings. Rather, the high density of multilayer (CNF/BA) coatings might be related to the formation of BA crystals that negatively affect the mechanical properties of the coating through brittleness and reduced chemical interactions between the BA crosslinker and nanocellulose (see Section 3.4). Alternatively, when comparing the mixed-layer (CNF + BA) coatings at different BA concentrations, the coatings with high-BA crosslinker concentrations provide the highest density and best mechanical resistance (see Section 3.4).

### 3.3. Physical Coating Properties

**Figure 8:**
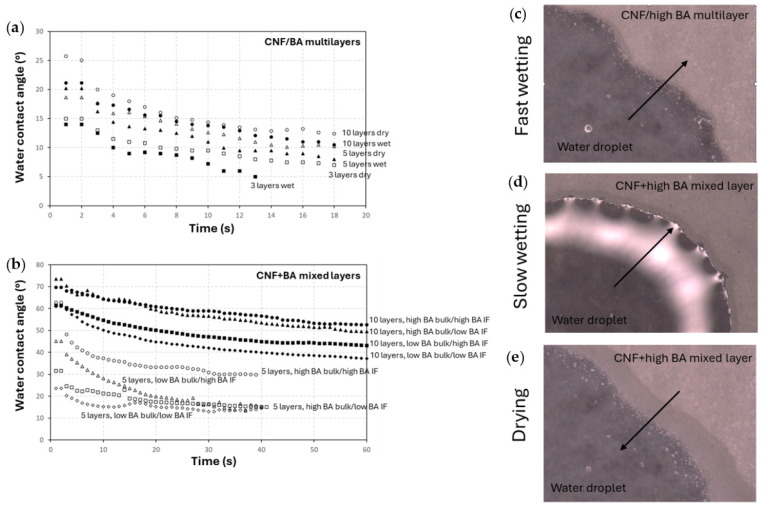
Water interaction of crosslinked coatings as determined from water contact angle measurements over time for (**a**) multilayer (CNF/BA) coatings and (**b**) mixed-layer (CNF + BA) coatings. Illustrations of the water droplet contact line with (**c**) advancing of a water droplet onto a multilayer (CNF/high BA) coating, (**d**) advancing of a water droplet onto a mixed-layer (CNF + high BA) coating, and (**e**) receding of a water droplet on a mixed-layer (CNF + high BA) coating with recovery of the coating structure during drying.

While the nanocellulose materials are strongly hydrophilic, their structural and dimensional stability in aqueous environments may be improved by crosslinking [13]. Hence, the effects of crosslinking of the nanocellulose coatings on the stability and interaction of water droplets deposited onto the surface were investigated by static water contact angle measurements to observe the spreading of the water droplets depending on the coating configuration and crosslinking (Figure 8).

The decrease in water contact angles over an observation time of 20 s was noticed because of spreading and penetration of the water on the coating (Figure 8a). The reference measurements of water droplets spreading on the substrates (glass, glass/PDA, and glass/PDA + BA) are presented in Supplementary Figure S6. The measurements for pure CNF coatings could not be performed as the water directly penetrates into the coating, while the crosslinked CNF coatings show retarded penetration of the water. The initial water contact angles upon direct contact (time < 1 s) for crosslinked coatings may increase due to roughness effects, but they are particularly higher for the mixed-layer coatings (CNF + BA), showing better water resistance. A direct relation between surface roughness and the initial water contact angle is not evident for the strongly hydrophilic coatings. It is obvious that the water stability over time increases for the coatings with a higher number of sprayed layers. For multilayer (CNF/BA) coatings, the rapid spreading of a water droplet over the coating is observed with a fast decrease in the water contact angle value over time. The high roughness of the multilayer (CNF/BA) coatings possibly relates to a higher surface porosity and easy water penetration. The multilayer coatings with intermediate drying and a high number of sprayed layers become more stable than the coatings deposited without intermediate drying, as the mechanical resistance of the coatings with intermediate drying also improved. For the mixed-layer (CNF + BA) coatings, the spreading and penetration of water over time were retarded with a higher stability for the mixed-layer coatings with high BA concentrations in the bulk and/or interface. The improvement in water stability is mostly determined by the high BA concentration as a crosslinker in the bulk (as related to the penetration of water in the bulk of the coating), while there additionally is a minor influence of the BA concentration in the interface (as related to the penetration of water in the interface). The stability and higher water contact angles for BA-crosslinked nanocellulose coatings are better as compared to previous studies reporting lower water contact angles for BA-crosslinked nanocellulose coatings on wood [56]. In parallel with the mechanical resistance of the mixed-layer (CNF + high BA) coatings, the coatings with high BA concentrations in the bulk or interface have higher water stability than the coatings with low BA concentrations, as related to the crosslinking density of the coatings. The fast wetting on multilayer (CNF/high BA) coatings can be seen from the progress of the water contact line (Figure 8b), while slow wetting on mixed-layer (CNF + high BA) coatings is observed as a slow advance of the water contact line over the coating and sharp definition of the water droplet border (Figure 8c). During drying of the water droplet, the contact line progressively retracts and the nanofibers re-arrange into a closed and dried coating (Figure 8d); the latter is an indication of structural recovery of the mixed (CNF + BA) coatings after water contact.

**Figure 9:**
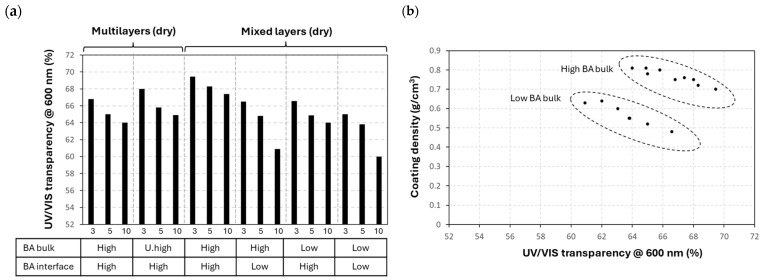
Transparency of crosslinked coatings as determined from UV/VIS spectroscopy: (**a**) transparency data summary for different coating configurations with 3, 5, and 10 sprayed layers, (**b**) relation between transparency and coating densities.

Although the thin nanocellulose coatings provide optical transparency owing to the size features of the cellulose nanofibers below the wavelength of light, the non-uniformities in the coatings, partially due to effects such as local organization of fibers into microscale entities, surface roughness, or porosity, can result in light-scattering effects and haze [76]. Therefore, the transparency loss of the coatings depending on the number of sprayed layers and internal crosslinking was quantified for the different coating configurations relative to the uncoated glass slide, both providing performance data and qualitatively relating to the internal homogeneity of the coating structure. The raw data of UV-VIS spectra and visual observation with an optical microscope are illustrated for some coating configurations (see Supplementary Figure S7), and (relative) transparency values at a selected wavelength (600 nm) are summarized for mixed and multilayer coatings with different BA concentrations as crosslinkers in the bulk or interface layer (Figure 9a). For both multilayer (CNF/BA) and mixed-layer (CNF + BA) coatings, the high concentrations of BA crosslinkers in the bulk of the coating provide higher transparency as compared to the low BA concentrations. Otherwise, the mixed-layer (CNF + high BA) coatings provide the highest transparency, reflecting better homogeneity as compared to the multilayer (CNF/BA) coatings. The transparency cannot solely be related to the coating thickness, but it relates best to the coating densities, with significant influence of BA crosslinker concentration in the bulk (Figure 9b). The higher density of the coatings through crosslinking intuitively implies the close interaction between the fibers, and hence there are likely fewer pores for light scattering. In comparison, the transparency of coatings with a sprayed PDA/BA interface layer on glass was inferior (see Supplementary Figure S8), in parallel with their heterogeneity and lower mechanical performance as indicated before, and this configuration was not further considered. As compared to the literature [77], a high transparency of >90% was only obtained for nanometer thickness of nanocellulose composite coatings with high density and a low fraction of nanovoids.

### 3.4. Chemical Coating Properties

**Figure 10:**
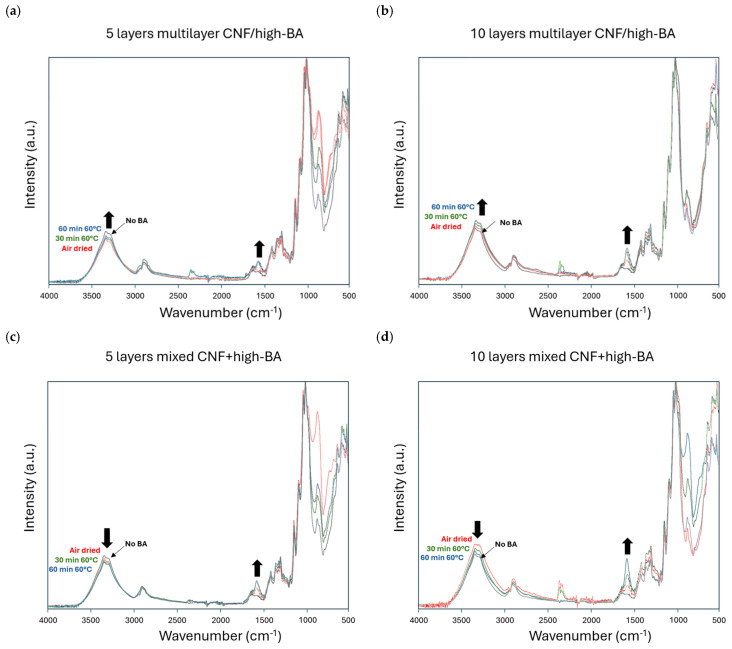
Infrared spectroscopy indicating the effects of drying on free hydroxyl groups for different coating configurations, including (**a**,**b**) multilayer (CNF/BA) coatings and (**c**,**d**) mixed-layer (CNF + BA) coatings. Arrows point towards interesting intensity changes (increasing or decreasing).

**Figure 11:**
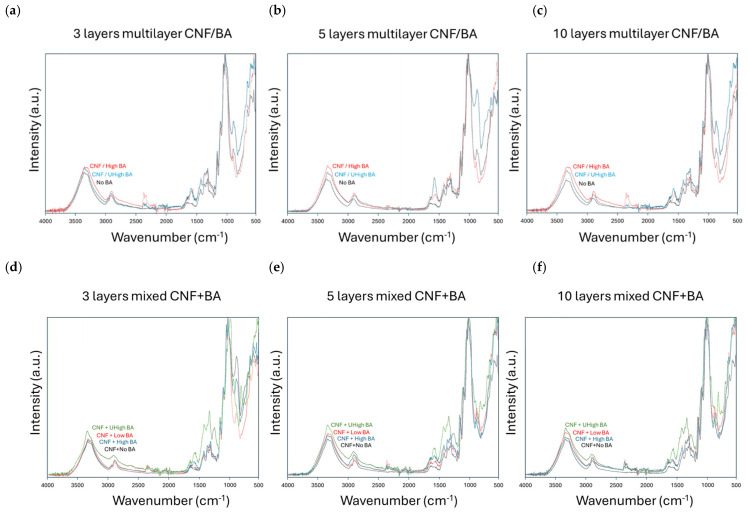
Infrared spectroscopy indicating the effects of BA concentration in the bulk for different coating configurations, including (**a**–**c**) multilayer (CNF/BA) coatings and (**d**–**f**) mixed-layer (CNF + BA) coatings.

**Figure 12:**
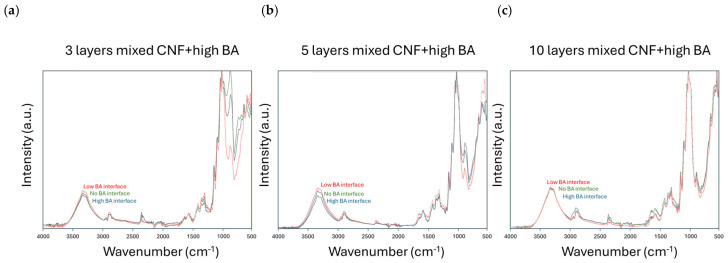
Infrared spectroscopy indicating the effects of BA concentration in the glass/PDA/BA interface layer for mixed-layer (CNF + high BA) coatings with different numbers of layers, including (**a**) 3 mixed layers, (**b**) 5 mixed layers, and (**c**) 10 mixed layers.

The chemical interactions between the CNF and BA in different coating configurations were studied by FTIR spectroscopy, revealing the effects of intermediate drying, the number of sprayed layers, and BA concentrations (Figure 10, Figure 11 and Figure 12). The characteristic spectral bands for boric acid are located at around 1400 cm^−1^ (B-O, B-O-B asymmetric stretching) and 1150 cm^−1^ (B-OH in-plane bending), which both strongly overlap with the characteristic fingerprint region of cellulose presenting the C-H and O-H bending bands (1450, 1426, 1365, 1335, 1314, 1280, 1247, 1203, and 1159 cm^−1^) and C-O vibration bands (1104, 1052, 1028, 1002, and 985 cm^−1^). The same bands in the range of 1420 to 1407 cm^−1^ were observed due to asymmetric B-O-C vibrations in the nanocomposite films with cellulose nanocrystals and BA, attributed to hybrid film formation [78]. Changes in the cellulose region at 1055 cm^−1^ and 1029 cm^−1^ were observed for some films of microcrystalline cellulose with a BA crosslinker, but no clear indication of interactions within this region was given as it might rather relate to the changes in cellulose structure [52]. Therefore, the maximum intensity of cellulose bands was used as an internal reference for our study, and the BA spectral bands can be related to the content of boric acid.

The spectral bands at 3800 to 3000 cm^−1^ contain complex contributions of O-H stretching for both cellulose and boric acid, with a maximum at around 3330 cm^−1^ for CNF and at around 3320 cm^−1^ for BA. While both peaks representing free hydroxyl cannot be separately resolved, they contribute to the overall intensity and width of the spectral hydroxyl band region and give a qualitative indication of relative differences in interaction between the components depending on the coating configuration. The same variations in intensities for this specific spectral band region were observed after the chemical modification of poplar wood flour with boric acid [79]. The interactions between free hydroxyl groups of BA and CNF are indicative of the crosslinking reaction forming a dense network through hydrogen interaction and possible covalent bonding under evaporation of the water. Depending on the composition and presence of hydroxyl groups in both components, an increase in free hydroxyl content is expected when mixing under conditions without interaction between the components. Alternatively, a reduction in the free hydroxyl content would imply the interaction and bonding between both components. According to the literature observations for CNF and CNC nanocellulose films [80], a proportional decrease in spectral bands of the nanocellulose can be expected with an increase in the BA ratio together with new peaks of BA.

For multilayer (CNF/high BA) and mixed-layer (CNF + high BA) coatings, the effects of drying are illustrated by comparing the spectra for coatings deposited under wet conditions (no intermediate drying) and dry conditions (with intermediate drying), as shown in Figure 10. The different effects of drying on the free hydroxyl band intensities (3800 to 3000 cm^−1^), relative to an increase in band intensities related to pure BA (1400 cm^−1^), are noticed for the conditions of air drying (no oven), mild drying (30 min, 60 °C), and intense drying (60 min, 60 °C). For multilayer coatings, the OH band intensity progressively increases depending on the more intense drying conditions for both thin (3- to 5-layer) and thick (10-layer) coatings, with the higher intensities related to the higher coating thickness. These trends signify no reduction in free hydroxyls due to unfavorable interactions or bonding in multilayer coatings: the results for weak chemical interaction are indeed in line with their relatively weak mechanical properties. The increase in band intensity for drying of the coatings with the same number of layers may be related to possible migration of free species towards the surface. When increasing the concentration of high BA towards ultra-high BA for the multilayer CNF/BA coatings, the reduction in OH band intensities upon drying indeed confirms the crosslinking (see Supplementary Figure S9), in parallel with the better mechanical performance. On the contrary, the drying of mixed-layer coatings indicates a progressive reduction in OH band intensities depending on the more severe drying conditions. The intense mixing of BA and CNF may indeed provide better conditions for crosslinking through the entire bulk of the mixed-layer coating at high BA concentrations. Alternatively, the interactions between BA and CNF in the multilayer coatings are restricted to the interfaces between both components, and the ultra-high BA concentrations are needed to ensure crosslinking. In conclusion, the first interpretation of interactions around free hydroxyl groups depending on drying conditions and coating configurations provides consistent data despite the relatively low concentrations of BA in the overall coating composition.

The chemical effects and interactions of variable BA crosslinker concentrations in the bulk of multilayer coatings (CNF/BA) and mixed-layer coatings (CNF + BA) are further detailed in the spectra for coatings with different numbers of layers, as shown in Figure 11. The spectra for multilayer coatings show consistent trends for all coatings with 3, 5, and 10 layers (Figure 11a–c), showing that the OH band intensities reduce for ultra-high-BA crosslinker concentrations as compared to high-BA crosslinker concentrations. This is a final confirmation that the multilayer coatings with ultra-high BA concentrations introduce better crosslinking as compared to the multilayer coatings with high BA concentrations, in parallel with the observed improvement in mechanical properties. The spectra for mixed-layer coatings with 3, 5, and 10 sprayed layers (Figure 11d–f) indicate that the OH band intensities are lowest for the compositions with high BA concentrations, which is proof of better crosslinking as compared to the composition with ultra-high BA concentrations. The higher intensities of free hydroxyl bands for the mixed-layer coatings with ultra-high BA concentrations can indeed be related to the reduced crosslinking interactions in parallel with the weaker mechanical properties observed before.

The chemical effects of the BA crosslinker concentrations in the interface of mixed-layer coatings (CNF + high BA) with the glass/PDA/BA substrate are illustrated in the spectra of Figure 12 for coatings with different thicknesses. The OH band intensities are compared for the coatings with 3, 5, and 10 mixed layers deposited on glass/PDA (no BA) and glass/PDA/BA substrates with low BA or high BA concentrations in the interface. The most differences in free hydroxyl group interactions are obviously observed for the thin coatings (3 or 5 layers), and no effects are seen for the thickest coatings (10 layers), as the interface interactions are not detected in the latter case. The relative changes in band intensities related to free hydroxyls are similar for the coatings with three or five sprayed layers and somewhat more pronounced in the latter case due to the higher thickness of coating present while still sensing the interactions with the interface layer. The relatively low spectral band intensities related to free hydroxyl groups for coatings deposited onto the glass/PDA/BA substrate with high BA concentrations as compared to low BA concentrations reflect a reduction through crosslinking between the mixed-layer coating and the interface with high BA concentrations, while the high concentrations of free hydroxyl groups for coatings deposited onto the interfaces with low BA concentrations apparently do not introduce the appropriate crosslinking. These observations were confirmed by previous mechanical testing, where the low BA concentration in the interface for mixed-layer coatings caused premature failure. In conclusion, the qualitative trends observed in interactions between BA and CNF through a reduction in free hydroxyl groups after crosslinking for given coating configurations are confirmed by the enhancement in mechanical properties of the coatings.

## 4. Conclusions

The mechanical robustness of sprayed nanocellulose coatings was enhanced through the design of different configurations with multilayer and mixed-layer coatings, including different concentrations of boric acid (BA) as an internal crosslinker and deposition on glass substrates with an adhesive interlayer of polydopamine/boric acid (PDA/BA). Depending on the selected coating configuration, the concentration of BA in the bulk is critical for favorable crosslinking, where the crosslinking between CNF and BA is demonstrated through a reduction in free OH groups for coatings with the best mechanical performance.

From the experimental evaluation of mechanical coating resistance, a balance between cohesive and adhesive strength has been determined by changing the BA crosslinker concentration in the glass/PDA/BA interface and bulk of the coating. First, an appropriate interface was developed with high BA concentrations required to enhance crosslinking between CNF and PDA/BA while preventing degradation of the PDA. Second, the concentration of the BA crosslinker in the bulk was modulated, resulting in multilayer coatings (CNF/ultra-high BA) with a maximum scratch resistance of 8 N, and mixed-layer coatings (CNF + high BA) with a maximum scratch resistance of 9 N.

The improvement in mechanical resistance of mixed-layer coatings (CNF + BA) is reflected in the higher density of coatings after crosslinking with BA, while the formation of single crystallites in CNF/BA multilayer coatings reflects inferior mechanical properties and brittleness. The crosslinking of mixed-layer coatings with BA is also confirmed by a retarded penetration of water and changes in transparency as a function of the coating density. The chemical interactions through hydrogen bonding after crosslinking of CNF and BA are qualitatively demonstrated, depending on the drying conditions and appropriate concentrations of BA crosslinker in the bulk and interface layer.

As presented in this work, the mechanical robustness of sprayed nanocellulose coatings can be ensured by crosslinking of multilayer and mixed-layer configurations, while the scalability of the spraying process enables its future application in more demanding industrial applications.

## Data Availability

The original contributions presented in this study are included in the article/Supplementary Material. Further inquiries can be directed to the corresponding author.

## Supplementary Materials

The following supporting information can be downloaded at https://www.mdpi.com/article/10.3390/polym17182451/s1: Figure S1: SEM evaluation of dried CNF Valida^®^ fine grade S191C; Figure S2: Mechanical resistance testing for multilayer coating (CNF/BA) and mixed-layer coating (CNF + BA) configurations deposited under “wet” conditions, i.e., without intermediate drying in between the different spraying layers: (a) multilayer coatings, (b) mixed-layer coatings. Only coatings with 5 and 10 sprayed layers are included because of inferior properties of coatings with only 2 sprayed layers; Figure S3: Mechanical resistance testing for mixed-layer coatings with a sprayed PDA layer without a BA crosslinker in the interface, and a sprayed PDA layer with a BA crosslinker in the interface; Figure S4: Detailed microscopic evaluation of crystallite structures for multilayer coatings (CNF/BA) deposited under wet conditions (without intermediate drying) or dry conditions (with intermediate drying); Figure S5: Coating deposition characteristics as a function of the number of sprayed layers, and final characteristics including a coating weight and coating density summary for different coating configurations with 3, 5, and 10 sprayed layers: extended graph with additional data for the sprayed adhesion layer; Figure S6: Reference measurements for water contact angles as a function of time for glass substrates, glass/PDA substrates, and glass/PDA/BA substrates; Figure S7: Illustration of raw data for transparency evaluation, including optical microscopy (left), and UV/VIS spectroscopy (right); Figure S8: Transparency of crosslinked coatings as determined from UV/VIS spectroscopy: extended graph with additional data for sprayed adhesion layer; Figure S9: Infrared spectroscopy for multilayer coatings (CNF/BA) with high BA and ultra-high BA concentrations.
