# Supplementary material for: Enhanced Mechanical Robustness of Sprayed Cellulose Nanofibril Coatings Through Internal Crosslinking with Boric Acid
Source: Polymers (Basel). 2025 Sep 10;17(18):2451. doi: 10.3390/polym17182451 (PMC12473480; doi:10.3390/polym17182451)

ENHANCED MECHANICAL ROBUSTNESS OF SPRAYED CELLULOSE NANOFIBRIL COATINGS THROUGH INTERNAL CROSSLINKING WITH BORIC ACID

Pieter Samyn[1]*, Patrick Cosemans[1], Erik Van der Eycken[2,3], and Guglielmo A. Coppola[2]

[1]Department of Innovations in Circular Economy and Renewable Materials, SIRRIS, Gaston Geenslaan 8, B-3001 Leuven, Belgium

[2]Laboratory for Organic & Microwave-Assisted Chemistry (LOMAC), Department of Chemistry, University of Leuven (KU Leuven), Celestijnenlaan 200F, B-3001 Leuven, Belgium

[3]Peoples' Friendship University of Russia (RUDN University), Miklukho-Maklaya Street 6, 117198 Moscow, Russia

*Correspondence: pieter.samyn@sirris.be

Supplementary information

Supplementary Information S1.

The cellulose nanofibrils (CNF) of Valida® fine grade S191C (Sappi, Maastricht, The Netherlands) were delivered as an aqueous suspension with dry solid content of 3 wt.-%. The CNF (see Supplementary Information S1) was produced under industrial conditions of mechanical fibrillation at pilot scale of hardwood pulp, as patented [64]. Full characterization of the materials cannot be disclosed owing to proprietary rights.

Key characteristics of the CNF material include:

- CAS number 9004-34-6
- Zetapotential of the suspension (measured on Malvern Zeta Nanosizer at 0.1 wt.-%) = -30 mV
- Fiber diameter, 20 to 50 nm with mean value 30 nm.
- pH of suspension = 7.8
- Total Fines > 92%
- Aspect ratio L/D = 15 to 20.

Figure S1. SEM evaluation of dried CNF Valida® fine grade S191C.

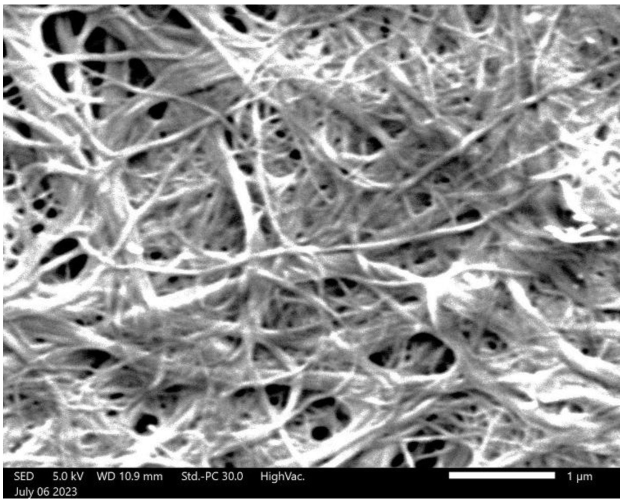

Supplementary Information S2.

Figure S2. Mechanical resistance testing for different coating configurations deposited under "wet" conditions, i.e. without intermediate drying in between the different spraying layers, (a) multilayer coatings (CNF/BA), (b) mixed layer coatings (CNF+BA). Only coatings with 5 and 10 sprayed layers are included because of inferior properties for coatings with only 2 sprayed layers.

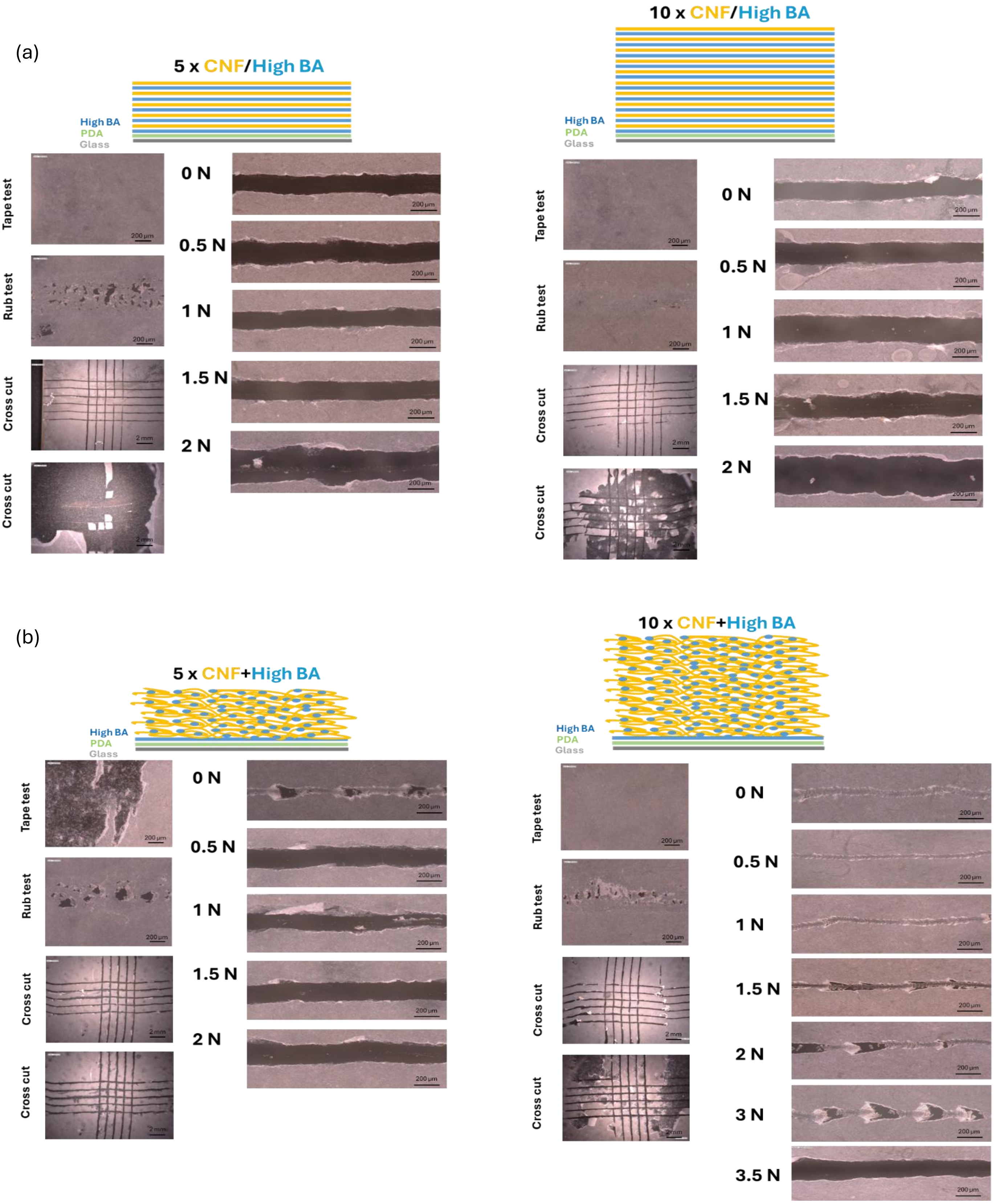

Supplementary Information S3.

Figure S3. Mechanical resistance testing for mixed coatings (CNF+high-BA)with sprayed PDA layer without BA crosslinker in the interface, and sprayed PDA layer with BA crosslinker in the interface

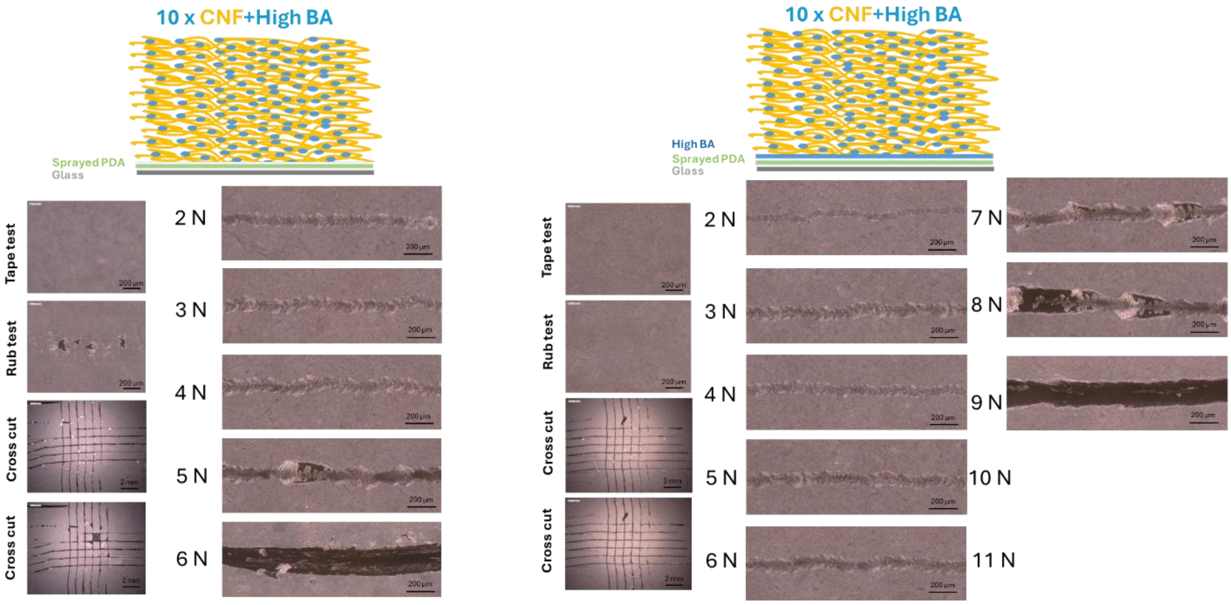

Supplementary Information S4.

Figure S4. Detailed microscopic evaluation of crystallite structures for multilayer coatings CNF/BA deposited under wet conditions (without intermediate drying), or dry conditions (with intermediate drying)

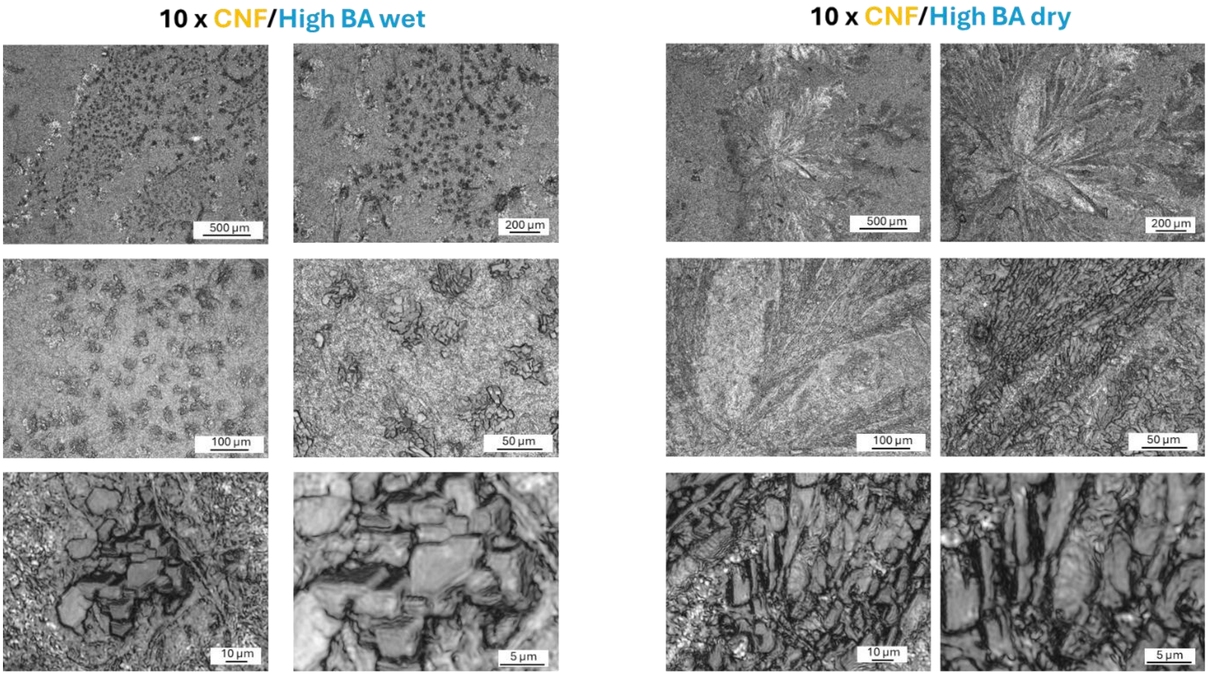

Supplementary Information S5.

Figure S5. Coating deposition characteristics as a function of number sprayed layers and final characteristics, including coating weight and coating density summary for different coating configurations with 3, 5, 10 sprayed layers: extended graph with additional data for sprayed adhesion layer.

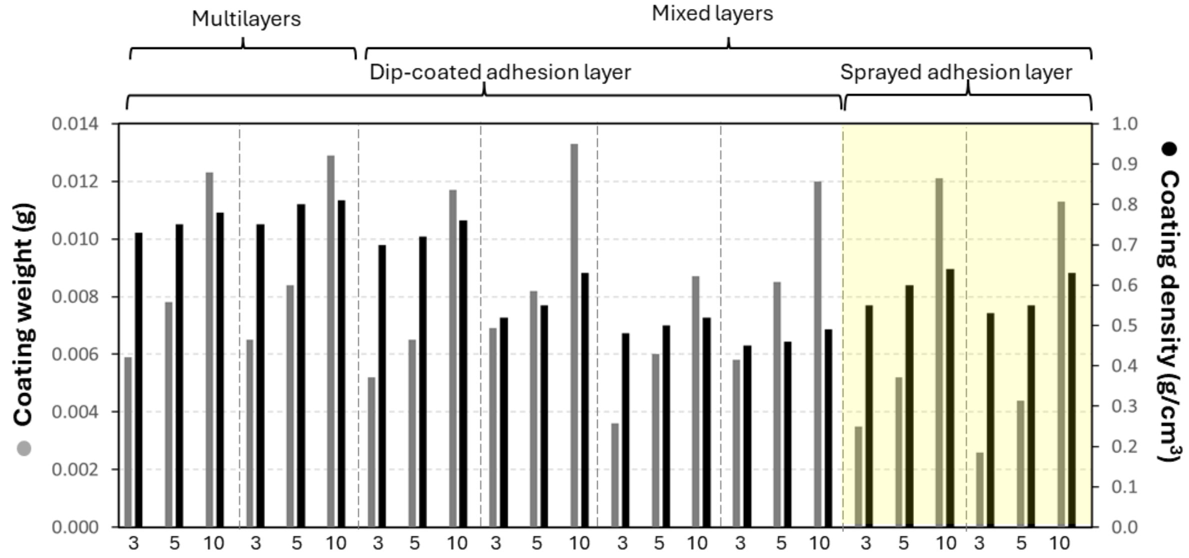

BA stack	High	U.high	High	High	Low	Low	High	Low
BA interface	High	High	High	Low	High	Low	High	High

Supplementary Information S6.

Figure S6. Reference measurements for water contact angles as a function of time for glass substrates, glass/PDA substrates, glass/PDA/BA substrates.

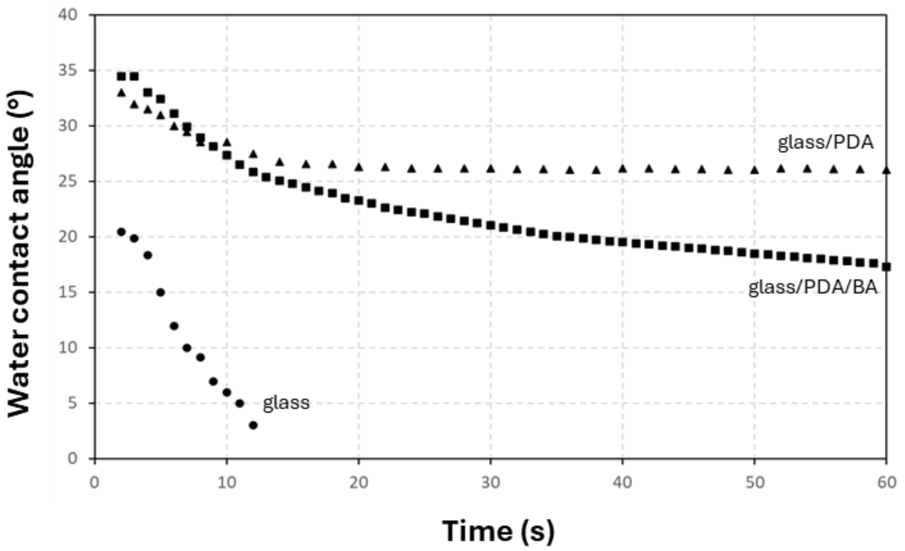

Supplementary Information S7.

Figure S7. Illustration of raw data for transparency evaluation, including optical microscopy (left), and UV/VIS spectroscopy (right).

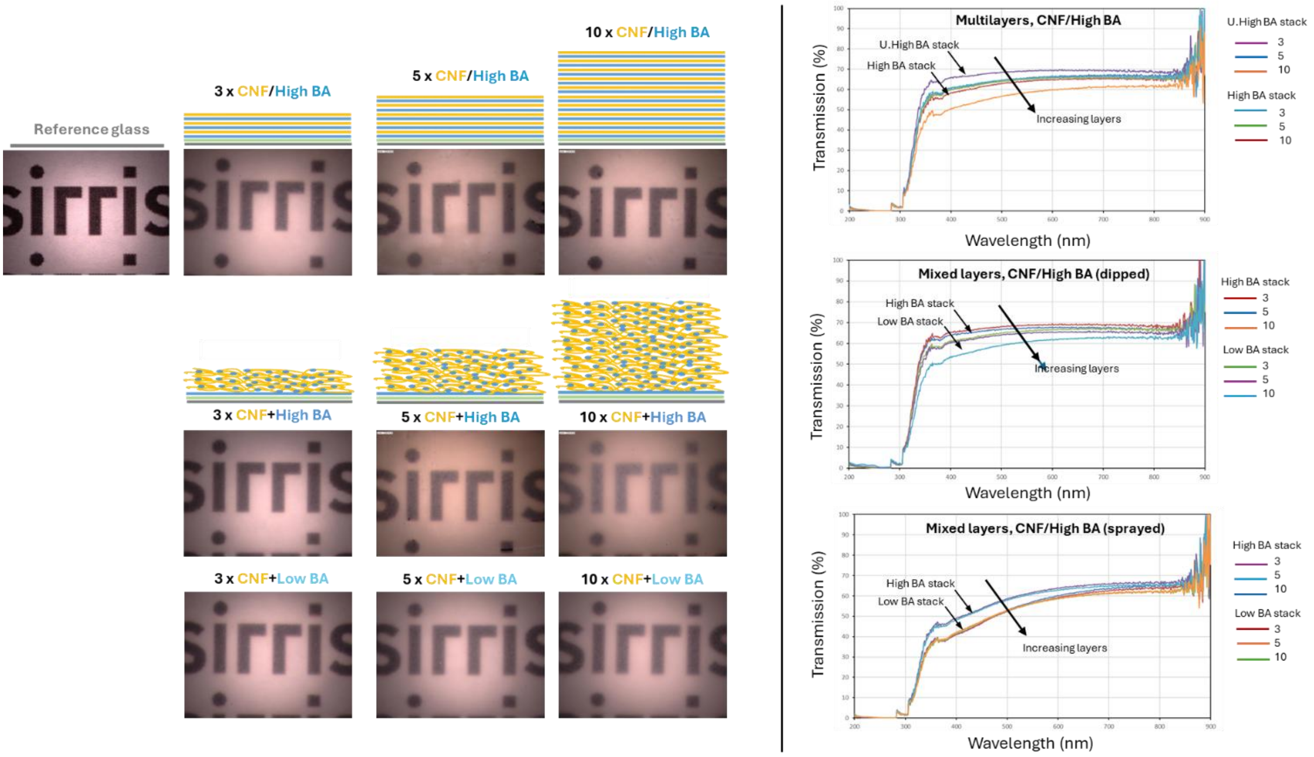

Supplementary Information S8.

Figure S8. Transparency of crosslinked coatings as determined from UV/VIS spectroscopy: extended graph with additional data for sprayed adhesion layer.

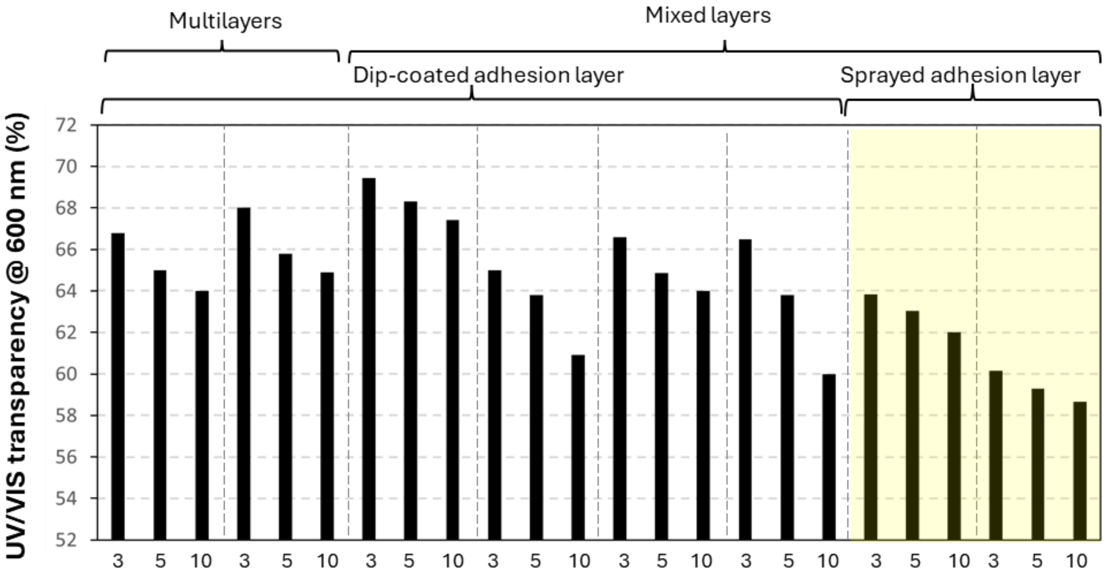

BA stack	High	U.high	High	High	Low	Low	High	Low
BA interface	High	High	High	Low	High	Low	High	High

Supplementary Information S9.

Figure S9. Infrared spectroscopy for multilayer coatings (CNF/BA) with high-BA and ultra-high BA concentrations.

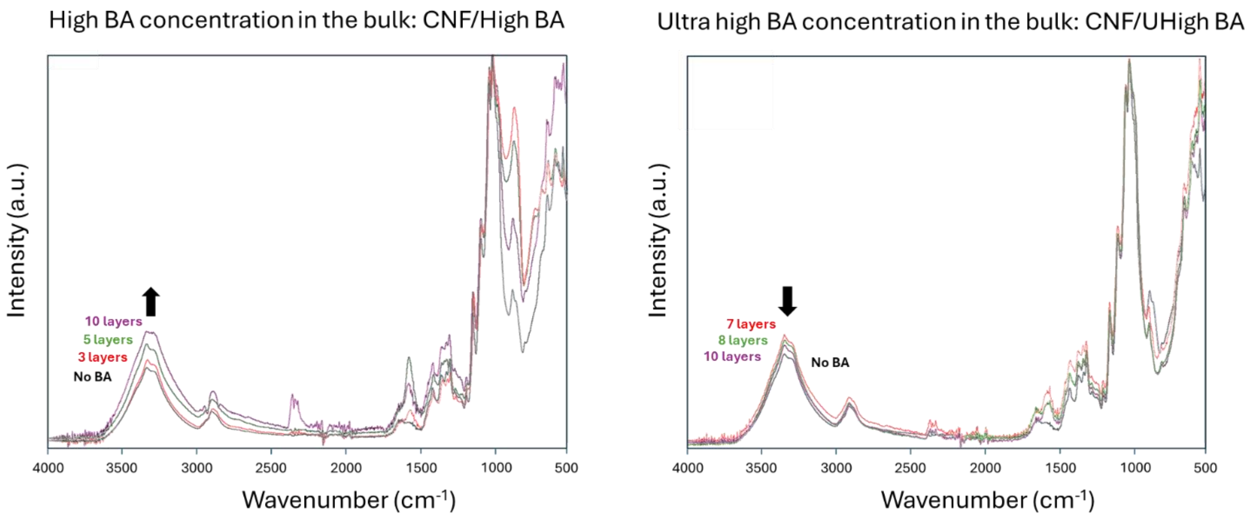

Supplement: Supplementary file 1 [file polymers-17-02451-s001.zip › polymers-3847319-supplementary.pdf]
